# Analysis of SMN protein in umbilical cord blood and postnatal peripheral blood of neonates with SMA: a rationale for prompt treatment initiation to prevent SMA development

**DOI:** 10.1186/s13023-025-03597-4

**Published:** 2025-02-28

**Authors:** Noriko Otsuki, Tamaki Kato, Mamoru Yokomura, Mari Urano, Mari Matsuo, Emiko Kobayashi, Kazuhiro Haginoya, Hiroyuki Awano, Yasuhiro Takeshima, Toshio Saito, Kayoko Saito

**Affiliations:** 1 Institute of Medical Genetics, Tokyo Women’s Medical University, 8-1 Kawadacho, Shinjuku-ku, Tokyo, 162-8666 Japan; 2 Department of Pediatrics, Gifu Prefectural General Medical Center, 4-6-1 Noisshiki, Gifu City, Gifu 500-8717 Japan; 3 Department of Pediatric Neurology, Miyagi Children’s Hospital, 4-3-17 Ochiai, Aoba-ku, Sendai City, Miyagi 989-3126 Japan; 4 Department of Pediatrics, Kobe University Graduate School of Medicine, 7-5-1 Kusunoki-cho, Chuo-ku, Kobe City, Hyogo 650-0017 Japan; 5 Organization for Research Initiative and Promotion, Tottori University, 36-1 Nishi-cho, Yonago City, Tottori, 683-8503 Japan; 6 Department of Pediatrics, Hyogo Medical University, 1-1 Mukogawacho, Nishinomiya City, Hyogo 663-8501 Japan; 7 Division of Child Neurology, Department of Neurology, National Hospital Organization Osaka Toneyama Medical Center, 5-1-1 Toneyama, Toyonaka City, Osaka 560-8552 Japan

**Keywords:** Spinal muscular atrophy, Survival motor neuron, Cord blood, SMN spot analysis, Imaging flow cytometry, Disease-modifying therapy, Newborn screening, Nusinersen, Onasemnogene abeparvovec, Risdiplam

## Abstract

**Background:**

Spinal muscular atrophy (SMA) is a severe genetic neuromuscular disease caused by insufficient functional survival motor neuron protein (SMN). The SMN expression level in the spinal cord is highest during the 2nd trimester of the foetal period. We previously reported the SMN spot analysis in peripheral blood using imaging flow cytometry (IFC) as a biomarker of functional SMN protein expression. In this study, we analysed neonatal cord blood, postnatal peripheral blood, and maternal peripheral blood in presymptomatic five infants whose sibling has type 1 SMA to estimate prenatal and postnatal SMN dynamics before the onset of severe SMA.

**Results:**

Data from 37 untreated patients with SMA showed that SMN-spot^+^ cells were significantly correlated with SMA clinical classification and the copy numbers of the *SMN2* gene. The range of values for cord blood, converted from each *SMN2* copy number statistics, was − 0.7 to + 2.0 standard deviation (SD) (0.1–24.0%) for SMN-spot^+^ cells in patients with SMA. Subsequent analyses of the peripheral blood of neonates ranged from − 0.8 to + 0.8 SD (0.4–15.2%). The analysis of each maternal blood, converted from carrier statistics, ranged from − 0.2 to + 2.4 SD (1.4–25.2%). A correlation was observed between the cord blood and maternal peripheral blood.

**Conclusions:**

This study suggests that the status of the motor neuron pool in the spinal cord can be presumed by cord blood SMN-spot^+^ cells and that SMN protein depletion determines the timing of disease onset. As the SMN spot analysis values tended to decrease with time after birth, they may eventually lead to the development of SMA. Furthermore, a correlation was found between the SMN spot analysis values of neonatal cord blood and maternal blood, which predicts disease severity after birth. In other words, the SMN protein supplied from the mother to the foetus may suppress the development of SMA in the infant at birth, and depletion of the SMN protein may occur after birth, causing the infant to develop SMA. Our findings demonstrated the effectiveness of newborn screening and the potential of maternally mediated treatment strategies by providing a rationale for prompt treatment initiation in SMA.

**Supplementary Information:**

The online version contains supplementary material available at 10.1186/s13023-025-03597-4.

## Background

Spinal muscular atrophy (SMA) is an autosomal recessive neuromuscular disorder associated with lower motor neuron degeneration due to the loss of spinal anterior horn cells. This is caused by deletions or mutations in the *survival motor neuron 1* gene (*SMN1*), which encodes the SMN protein (SMN) [1]. The pathological condition of the SMA is classified into types I to IV based on the age of disease onset and achievement of motor milestones [2].

SMN is ubiquitously expressed in eukaryotic somatic cells which is primarily involved in RNA metabolism and regulates cell biogenesis [3]. Various studies have reported that SMN deficiency causes motor neuron degeneration and proximal muscle weakness [4–6]. In humans, when *SMN1* is defective, the full-length (FL)-SMN protein is produced exclusively from *SMN2*, a paralogue of *SMN1*. However, *SMN2* has a C–T replacement in exon 7 that does not affect the amino acid sequence but causes exon 7 to be skipped during alternative splicing [7, 8]. As a result, about 90% of SMN produced from *SMN2* are not fully functional [9]. The copy number of *SMN2* in the human genome varies and most patients have two or more copies of *SMN2*. A high copy number of *SMN2* can increase the production of fully functional SMN and reduce disease severity [10].

Several motor function scales have been used for the clinical evaluation of SMA. The selection and combination of these scales should change depending on the age and severity of patients with SMA. Moreover, the evaluator may influence the assessment criteria. Therefore, an objective molecular biomarker is essential to determine the continuation of SMA treatment or therapeutic efficiency in all patients with SMA. Potential molecular biomarkers, such as serum cathepsin D, creatinine, creatine kinase, neurofilament, and microRNAs, have been examined [11, 12]. Among these, the SMN protein is a crucial molecule, and various types of quantitative analyses have been reported. However, it has not yet been demonstrated as a highly sensitive and versatile marker.

In 2018, we developed the SMN spot analysis as a new biomarker for SMA using imaging flow cytometry (IFC) [13]. We analysed the percentage of SMN-spot^+^ CD33^++^ cells present in peripheral blood mononuclear cells (PBMC). Our findings revealed significant differences in the percentage of SMN-spot^+^ cells in the CD33^++^ cell fraction between the SMA and non-SMA groups. We also demonstrated a partial correlation between SMN spot analysis values and clinical information among patients. Furthermore, we demonstrated the co-localisation of the SMN spot with the functional molecule, which enabled us to analyse the expression levels of functional SMN proteins instead of merely comparing SMN expression. In our previous study, we explained the reason for selecting CD33^++^ cells as the target for our analysis.

In mice, various studies have shown that SMN expression is necessary for embryo–foetal development. However, it is difficult to validate multiple human spinal cord tissues from the prenatal to postnatal periods. In 2019, Ramos et al. conducted a quantitative analysis of the SMN protein and mRNA levels in the spinal cord and cortex tissues of 75 non-SMA and 16 SMA patients. This study confirmed the significance of SMN from the foetal stage to three months after birth and determined the period of highest demand of SMN [14].

We focused on SMN protein analysis in neonatal umbilical cord blood. We collected umbilical cord blood immediately after birth from the placenta of neonates, prenatally diagnosed with SMA by amniotic fluid or chorionic villus sampling, in five families whose previous child had type I SMA. Additionally, we examined the transition of functional SMN expression from the neonatal to the postnatal period in the peripheral blood. We also performed SMN spot analysis on their maternal peripheral blood. In this observational study, examinations were considered less invasive as we used the umbilical cord blood and peripheral blood. Disease-modifying therapy (DMT) intervention was planned to begin at 37–41 weeks of gestation at full-term delivery. Based on these results, we clarified a rationale for prompt initiation of DMT [15, 16]. Furthermore, we discuss possible therapeutic principles.

## Methods

### Study population

Peripheral blood was collected from 37 untreated patients and 18 parents, including five mothers of neonates. The 37 untreated patients were in the age range of 0.1–62 years. Details of the 37 patients are listed in Supporting information along with the analysis values (Additional file 1). The clinical classification was based on a study by Kaneko et al. [17]. Within the parental cohort, ten were mothers, and eight were fathers. Supplementary Information (Additional file 4) includes analyses of the carrier mothers and clinical information related to their children with SMA. Blood samples from the mothers were collected at normal conditions after delivery and completion of the lactation period.

In families with firstborn type I SMA, the prenatal diagnosis was performed during subsequent pregnancies under genetic counselling of clinical geneticists and certified genetic counsellors. Postnatal genetic evaluations definitively ascertained that the five neonates possessed null copies of *SMN1* exons 7 and 8. Each neonate was designated as Case 1 through 5, and the clinical profiles of their siblings are described in Table 1. Umbilical cord blood was harvested from the placenta of each neonate immediately postpartum, followed by peripheral blood sampling at intervals of several months. Genetic scrutiny of the patients with SMA involved the examination of exons 7 and 8 copy numbers on both *SMN1* and *SMN2* genes at the Institute of Medical Genetics, Tokyo Women’s Medical University (TWMU: Shinjuku, Tokyo, Japan), which is an antecedent to diagnostic confirmation. Patients were recruited from TWMU Hospital under Institutional Review Board-approved protocols, specifically No. 4786 and No. 5639. All procedures adhered to the ethical principles of the Declaration of Helsinki. Prior to engagement, written informed consent was meticulously acquired from the participants and/or their legal guardians in accordance with institutional guidelines.

**Table 1:** Demographics of patients

Pathological condition										
	Family 1		Family 2		Family 3		Family 4		Family 5	
	Sibling 1	Case 1	Sibling 2	Case 2	Sibling 3	Case 3	Sibling 4	Case 4	Sibling 5	Case 5
Sex	Male	Male	Female	Male	Female	Female	Male	Male	Male	Female
SMA type	Ia	–	Ia	–	Ia	–	Ib	–	Ib	–
Copy number of *SMN2* (exon 7)	2	2	2	2	2	2	3	3	3	3
Gestational age at delivery	–	37w1d	–	37w0d	–	38w0d	–	38w3d	–	38w1d
Symptoms at birth	Diminished fetal activity	Deep tendon reflex( −)	–	Asymptomatic	–	Asymptomatic	–	Asymptomatic	–	Asymptomatic
Symptoms of the onset	Not acquired head control	–	Not acquired head control	–	Not acquired head control	Frequent stumbling	Loss of head control	–	Loss of head control	Frequent stumbling
Onset of SMA	At birth	–	< 0y6m	–	0y4m	2y7m	0y4m	–	0y4m	3y11m
Procedure	Tracheostomy Gastrostoma	–	–	–	Tracheostomy Gastrostoma	–	–	–	–	–

**Disease-modifying therapy (DMT)**										
DMT initiation (gestational age)	–	37w3d	–	37w2d	–	40w2d	–	40w1d	–	41w0d
Nusinersen	4y1m –5y0m	3d	0y6m –1y0m	3d	1y7m	–	–	–	3y3m –ongoing	–
Onasemnogene abeparvovec	–	11d	1y2m	15d	–	17d	–	15d	–	21d
Risdiplam	–	0y8m –ongoing	–	–	6y7m –ongoing	2y9m –ongoing	–	4y0m –ongoing	–	4y0m –ongoing
Present status	Deceased at 5y2m	Pulling to stand	Sitting without support	Normal motor function	Not acquired head control (bedridden)	Climbing stairs with support	Deceased at 1y4m	Normal motor function	Loss of head control (wheelchair)	normal motor function
Last observation (age)	5y2m	1y11m	3y4m	0y5m	6y3m	4y3m	1y4m	4y2m	8y4m	4y4m

### Cells

The peripheral blood samples were collected in sodium heparin tubes. Blood samples were maintained at ambient temperature (15–26 °C) and shielded from light until processing. The staining procedure for PBMC underwent slight modifications from a previously published protocol [13]. On the day following blood collection, 1.5 mL of the blood sample was dispensed into a 15-mL conical tube. As an Fc-receptor-blocking, reagent for human blood, 30 μL of normal human Ig solution (Clear Back, MTG-001, MBL, Nagoya, Japan) was added to the dispensed blood and incubated for 15 min at room temperature. Subsequently, fluorochrome-conjugated monoclonal antibody (mAb) against CD33 was added to identify specific cell fractions. After incubation for 30 min at room temperature, blood samples were treated with 10 mL of pre-warmed Lyse/Fix Buffer (BD Biosciences, San Jose, CA, USA) and incubated at 37 °C in a water bath for 10 min. The cells were centrifuged at 900×*g* and the supernatant was aspirated. Cells were washed with 15 mL of PBS (−) (Ca^2+^ and Mg^2+^ free phosphate buffered saline) on time, and the supernatant was removed. The fixed and washed cells were permeabilised in two steps. First, cells were incubated with 1 mL of 0.2% TritonX-100/1% bovine serum albumin (BSA)/PBS (−) at room temperature for 5 min. Immediately after incubation, 3 mL of 1% BSA contained Perm/Wash Buffer (BD Biosciences) was added. The cell suspension was then incubated at room temperature in the dark for 60 min. After permeabilisation, the cells were washed with a staining buffer. The composition of the staining buffer was 0.05% Tween 20 and 1% BSA, containing Perm /Wash Buffer. Subsequently, the cell concentrations were counted and adjusted, and 1 × 10^6^ cells in 50 μL of staining buffer were dispensed into each of the two 1.5-mL microtubes. The cells were stained with mAbs against human SMN or isotype control and incubated for 45 min at room temperature. After incubation, the cells were washed thrice with staining buffer and finally with PBS for protein removal. Washed cells were resuspended in 50 μL of PBS (−) and analysed using imaging flow cytometry.

### Antibodies

The Alexa Fluor 488 (AF488)-conjugated anti-SMN mAb (2B1, murine IgG1κ) was purchased from Novus Biological (Littleton, CO, USA). Peridinin-Chlorophyll-Protein/Cyanin 5.5 (PerCP/Cy5.5) -conjugated anti-CD33 mAb (WM53, murine IgG1κ) and AF 488-conjugated murine IgG1κ (MOPC21) were purchased from BioLegend (San Diego, CA, USA).

### Imaging flow cytometry

Stained cell suspensions (2 × 10^7^ cells/mL) in 50 μL of PBS (−) were acquired using imaging flow cytometry (ImageStreamX Mark II, Cytek biosciences, Fremont, CA). The system was equipped with two cameras, a 60 × magnification objective lens (1 pixel = 0.3 μm × 0.3 μm), and 12 detection channels controlled by INSPIRE^®^ software (Cytek Biosciences). The raw image files were analysed using IDEAS^®^ software (Cytek Biosciences). Samples with a bright-field (BF) area of less than 60 pixels (5.4 μm^2^) were gated to eliminate debris. A minimum of 5000 cells within the CD33^++^ fraction were collected per sample. The phenotypes of CD33^++^ cells and details of the SMN spot analysis have been described previously [13].

The cell population strongly expressed CD33 in the umbilical cord blood at significantly higher side scatter (SSC) levels than in the lymphocyte fraction (Additional file 2B), similar to the distribution in PBMC, as we previously reported (Additional file 2A). We extracted the image data of the CD33^++^ fraction from the whole PBMC data to discriminate the target fraction that strongly expresses CD33 than the weakly expressed CD33. The population of CD33^++^ cells contained 85–90% CD14^+^ cells and 4–6% lineage marker^−^ (Lin^−^) cells. We regarded the CD33^++^ fraction as the CD14^+^ monocyte-dominated fraction. For an objective evaluation, an analysis was performed using the SMN spot detection algorithm constructed based on the ratio of fluorescence intensity per pixel.

### Statistical analysis

The Shapiro–Wilk normality test was performed to determine the normality of the data. The trend of continuous variables was assessed using Spearman's rank correlation test (**p < 0.01) and the Jonckheere-Terpstra test (*p < 0.05, **p < 0.01). The Mann–Whitney U test, Steel–Dwass test, and Kruskal–Wallis test were used to compare differences in each SMA group. All data were analysed using EZR statistical software (Easy R version 1.55), which is available online (https://www.jichi.ac.jp/saitama-sct/SaitamaHP.files/statmed.html) [18]. Statistical analysis of a limited number of cases was performed using G*Power (GPower 3.1), which is available online (https://www.psychologie.hhu.de/arbeitsgruppen/allgemeine-psychologie-und-arbeitspsychologie/gpower) [19].

## Results

### The correlation between the SMN spot analysis value and the clinical information in untreated 37 SMA patients

**Fig. 1:**
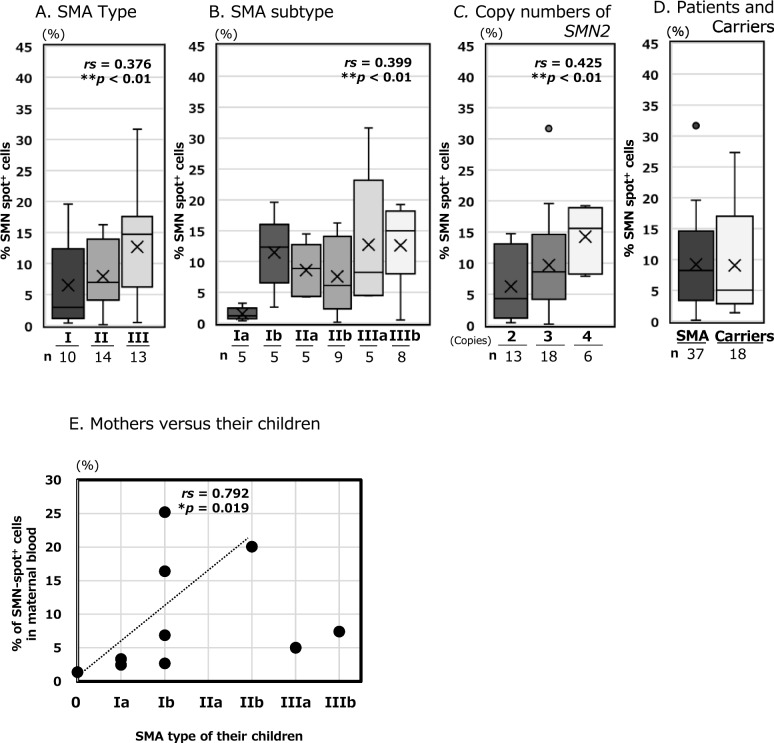
The survival motor neuron (SMN) spot analysis among the spinal muscular atrophy (SMA) patients and carriers. (**A**–**C**) Percentage of SMN-spot^+^ cells (y-axis) with (**A**) SMA typing, (**B**) clinical subtype, and (**C**) copy number of *SMN2* (x-axis). Details of each boxplot are given below the plot. The numerals given below the boxplot represent the number of participants (n). All patients are disease-modifying therapy (DMT)-naïve. (n = 37). (**D**, **E**) The percentage of SMN-spot^+^ cells (y-axis) in carrier group. (**D**) Mothers (ten) and fathers (eight) of SMA patients volunteered. (**E**) The relationship between SMN spot values in ten mothers (y-axis) and SMA severity in their children (x-axis). Statistical analysis was performed using Spearman’s rank correlation coefficient (two-tailed). Correlation coefficients (*rs*) and significance levels (*p*) are shown in the upper right side of the boxplots. (**p < 0.01, *p < 0.05). Details of the 37 patients with SMA and the 18 of carriers are presented in Additional file 1 and 4. Statistical analysis by the classification of patients with SMA and genetic carriers compared to historical control data is detailed in Additional File 3

First, we analysed untreated 37 patients to examine the correlation between disease severity and the analytical values. Information about the 37 patients and their analytical values can be found in Additional file 1. Figure 1A–C shows the distribution of the percentages of SMN-spot^+^ cells in 37 patients categorised by clinical classification and *SMN2* copy number. A positive correlation was observed between SMN spot analysis values and the two types of clinical classification (I–III and Ia–IIIb) (Fig. 1A, B). Spearman’s correlation coefficient (*rs*) was 0.376 for types I–III (**p < 0.01, Fig. 1A) and 0.399 for types Ia–IIIb (**p < 0.01, Fig. 1B). Moreover, a slightly stronger correlation was observed between SMN spot analysis values and *SMN2* copy numbers, with *rs* = 0.425 (**p < 0.01) (Fig. 1C). The results demonstrated that the data of SMN spot analysis correlated with the *SMN2* copy number and clinical classification, and details of each statistic are shown in Additional file 3A-C.

### Three-group comparison of SMN spot analysis among patients, the carriers, and control

Next, we performed SMN spot analysis on the peripheral blood of 18 parents whose children had two, three, or four copies of *SMN2* (Fig. 1D and E, Additional file 4). The median ± standard deviation (SD) value of the 18 carriers was 5.1% ± 8.5 (Fig. 1D). Similarly, the median value for the 37 patients group containing two, three, or four copies was 8.3% ± 7.1. According to the previously reported value of 14 cases of the control group (28.6% ± 8.4), the carrier group had a significantly lower percentage of SMN-spot^+^ cells than the controls (**p < 0.01). Furthermore, no significant differences were observed in the analysed values between the carrier and patient groups. We presented the median and SD within this article as percentages of SMN-spot^+^ cells. The information on the 37 patients can be found in Additional file 1, while the analytical value of carriers and clinical information about those children is available in Additional file 4.

To examine the variation among carriers, we investigated the relationship between the mother's SMN spot analysis value and the SMA severity of the child (Fig. 1E). This result showed no correlation among the 10 mother–child pairs. In an ongoing study, we have observed a tendency for the SMN spot analysis value to be inversely correlated with age in the control group (data not shown). To refine our analysis, we excluded mothers over the age of 40, resulting in a sample of eight mothers aged 25 to 39 years. In these cases, we identified a significant correlation (*rs* = 0.792, *p* = 0.019). In addition, these eight cases were mothers whose children were diagnosed with severe SMA types 0 to II (see fitted curve in Fig. 1E).

### The SMN spot analysis in neonatal umbilical cord blood and postnatal peripheral blood

To estimate functional SMN during the foetal period, SMN spot analysis was performed using umbilical cord blood derived from the placenta of five neonates (Case 1–5, Table 1). The delivery situation and DMT administration to the five neonates, along with the clinical history of their affected older siblings, are detailed in Table 1. At birth, Case 1 showed negative deep tendon reflexes (DTR (−)), which was suspected to be a symptom related to SMA, but no other SMA findings were observed. The percentages of SMN-spot^+^ cells in the cord blood of Case 1, 2, and 3 were 0.1, 0.6, and 13.1%, respectively. Case 1, 2, and 3 belonged to the 2 *SMN2* copies group. Therefore, when the deviation from the median of the 13 patients in the 2 *SMN2* copies group in Fig. 1C is converted into an analytical value using SD (Z-score), Case 1, 2, and 3 showed − 0.7 SD, − 0.6 SD, and + 1.6 SD, respectively. Cases 4 and 5 belonged to the 3 *SMN*2 copies group. Cases 4 and 5 were 3.6 and 24.0%, respectively. The Z-score of 18 patients in the 3 *SMN2* copies group in Fig. 1C, Case 4 and 5 were − 0.6 SD and + 2.0 SD, respectively. Although DTR (−) was assessed only in Case 1, all five neonates did not show any clinical symptoms of SMA.

Next, postnatal changes in the percentage of SMN spot^+^ cells in five neonates were monitored using peripheral blood. The second SMN spot analysis was performed at the earliest of 7-day-old (Case 4 and 5) and the latest of 59-day-old (Case 3). In the 2 *SMN2* copies group, Case 1 was 0.9% (− 0.6 SD), Case 2 was 0.4% (− 0.7 SD), and Case 3 was 3.0% (− 0.2 SD). In the 3 *SMN2* copies group, Case 4 was 2.5% (− 0.8 SD), and Case 5 was 15.2% (+ 0.8 SD) (Table 2).

**Table 2:** Details of analysis data in five patients and their mothers

	*SMN2* 2 copies			*SMN2* 3 copies	
(%SMN spot^+^ cells)	Case 1	Case 2	Case 3	Case 4	Case 5
*Patients*					
**Cord blood**	0.1%	0.6%	13.1%	3.6%	24.0%
Deviation from SMA median (σ_1_, σ_2_)	− 0.7 σ_1_	− 0.6 σ_1_	+ 1.6 σ_1_	− 0.6 σ_2_	+ 2.0 σ_2_
**Peripheral blood** ^a^	0.9%*	0.4%*	3.0%*	2.5%	15.2%
Deviation from SMA median (σ_1_, σ_2_)	− 0.6 σ_1_	− 0.7 σ_1_	− 0.2 σ_1_	− 0.8 σ_2_	+ 0.8 σ_2_
Blood sampling (age in days)	43d	29d	59d	7d	7d
*Mothers*					
**Peripheral blood**	1.4%	2.5%	3.4%	2.7%	25.2%
Deviation from Carrier median (σ_3_)	− 0.4 σ_3_	− 0.3 σ_3_	− 0.2 σ_3_	− 0.3 σ_3_	+ 2.4 σ_3_

^a^The data of patients’ peripheral blood were obtained from the second postnatal blood drawing

^*^Blood sampling day of Case1 (43d), Case 2 (29d), and Case 3 (59d) were after initiation of disease-modifying therapy (DMT). Details are described in Table 1 and below,

Case 1 received nusinersen at 3-day-old and onasemnogene abeparvovec at 11 days of age

Case 2 received nusinersen at 3-day-old and onasemnogene abeparvovec at 15 days of age

Case 3 received onasemnogene abeparvovec at 17 days of age

Numerical details are shown in the Additional file 3C, Median ± SD (σ) are as below

σ_1_: 2 *SMN2* copies group; 4.3% ± 5.7, σ_2_: 3 *SMN2* copies group; 8.6% ± 7.8, σ_3_: Carrier group; 5.1% ± 8.5

In Cases 1, 2, and 4, the umbilical cord blood analysis values were lower than the median values for each group with the same *SMN2* copy number, and the subsequent peripheral blood analysis also showed lower values. In contrast, in Cases 3 and 5, the cord blood analysis values were higher than the median values of each group by + 1.6 SD and + 2.0 SD. However, in the second peripheral blood analysis, Case 3 was below the median, − 0.2 SD, and Case 5 was + 0.8 SD. Both analysis values showed a decline compared to the umbilical cord blood values.

**Fig. 2:**
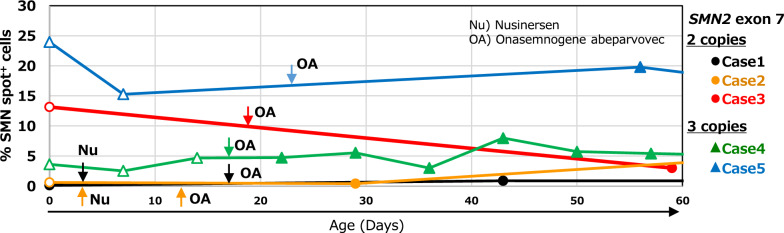
The survival motor neuron (SMN) spot analysis in the five spinal muscular atrophy (SMA) neonates. The percentage of SMN-spot^+^ cells (y-axis) is based on the number of days since birth (x-axis). The first point is the date of birth and the day the cord blood was analysed. Arrows indicate the date of nusinersen (Nu) or onasemnogene abeparvovec (OA) administration. Open symbols represent the analysis in the untreated period, and closed symbols represent the analysis after the start of disease-modifying therapy (DMT). The dates of DMT initiation are listed in Table 1

These results show that SMN spot analysis values may be higher at birth in neonates and those values may decrease with time after birth. Figure 2 shows the procedures of the postnatal DMT and the changes in the analysis values up to 60 days. (Table 1).

### The SMN spot analysis on the mothers of the SMA neonates

In parallel with the neonates, SMN spot analysis was performed on the mothers of five neonates. All five mothers were carriers and non-SMA. Table 2 shows the percentage of SMN-spot^+^ cells and Z-scores converted from the median and SD of the carrier group (Table 2). The mothers of Cases 1, 2, 3, 4, and 5 showed 1.4% (− 0.4 SD), 2.5% (− 0.3 SD), 3.4% (− 0.2 SD), 2.7% (− 0.3 SD), and 25.2% (+ 2.4 SD), respectively. The percentage of SMN-spot^+^ cells with Z-scores in the neonates' and mothers' blood samples is summarised in Table 2.

### Comparison of neonatal umbilical cord blood and maternal blood

**Fig. 3:**
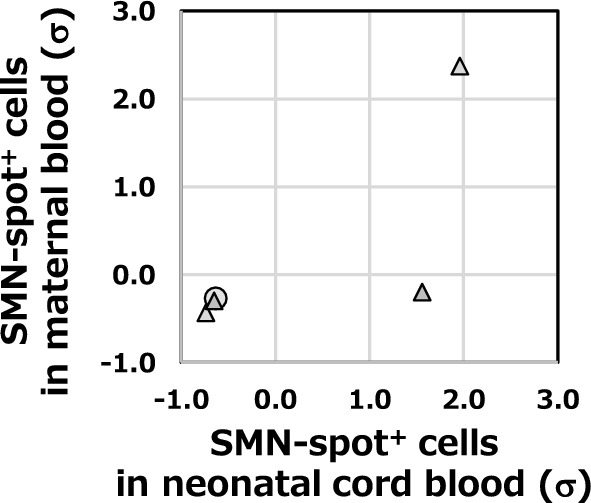
Correlation between maternal peripheral blood and neonatal cord blood. The SMN spot analysis data for maternal peripheral blood and neonatal cord blood shown in Table 2 are presented as scatter plots to examine the correlation between mothers and children. For each data point, the deviation from the median of the group to which it belongs is shown based on the standard deviation (Z-score), and mother-neonate pairs were compared. The x-axis shows the Z-score of neonatal cord blood. The y-axis shows the Z-score of maternal peripheral blood. Correlation analysis is described in the Discussion section

During pregnancy, it has been known that the transport of substances or molecular crosstalk occurs between the mother and foetus. Therefore, we investigated whether maternal substances affected SMN protein in the foetus. Based on Table 2, we examined whether there was a correlation in SMN spot analysis values between neonates and mothers. Case 1, 2, and 4 had below-median cord blood analysis results. The analysis values for the mothers’ blood were also below the median of the carrier. In contrast, Cases 3 and 5 had cord blood analysis values higher than the median. The analysis values for the mothers' blood tended to be higher than those of the other mothers. Therefore, we plotted neonatal umbilical cord blood and maternal peripheral blood for the five neonate-mother pairs using the Z-score specific for each group (Fig. 3). The five neonate-mother pairs were tested for correlation using Spearman’s rank correlation coefficient. The positive correlation was *rs* = 1.00 (*p = 0.017).

## Discussion

### The necessity of biomarkers for SMA

Objective molecular biomarkers are needed to determine the therapeutic efficiency of SMA, regardless of changes in growth or pathological conditions. Multi-omics analysis, such as proteome and transcriptome analysis, has progressed in recent years, leading to the identification of several potential biomarkers.

Among them, subunits of neurofilament (NF), such as neurofilament light chain (Nf-L) or phosphorylated neurofilament heavy chain (pNf-H), have shown promise in evaluating the degree of neurodegeneration, inflammation, and demyelination in the SMA. Several meta-analyses have shown that Nf-L is significantly elevated in several neurodegenerative diseases, including Charcot-Marie-Tooth disease, Guillain–Barre syndrome, Parkinson disease, and multiple sclerosis [20, 21]. In infants with type I SMA under the age of 18 months, plasma pNf-H was significantly higher than in non-SMA infants of the same age, and a 70% decrease was observed at two months with nusinersen administration [22]. In addition, Nf-L levels decreased in 12 children with type I SMA after receiving five or more doses of nusinersen [23]. However, in type II and type III patients or in adolescents and adults with SMA, neither pNf-H nor Nf-L was affected by nusinersen [24–28]. Therefore, NF may be applicable as a biomarker only in partial patients with SMA.

On the other hand, SMN has also been proposed as a biomarker through various quantitative analyses such as Enzyme-linked immunosorbent assay (ELISA) [29, 30], Homogeneous time resolved fluorescence (HTRF) [31], and Electrochemiluminescence (ECL) [32, 33]. However, even if SMN are quantified in whole PBMCs, it may be difficult to evaluate them as sensitive biomarkers because PBMCs are constructed from heterogeneous cell populations with different SMN expression levels. In a previous report [13], we mentioned that SMN spot analysis would detect the intranuclear aggregation of SMN proteins and their association with other functional molecules. Our previous report suggested that it may be an effective biomarker for SMA, regardless of age or severity. However, it is important to note that CD33^++^ cells are the lymphocyte fraction and not nerve cells. While orally administered risdiplam affects SMN expression in peripheral blood [34], nusinersen and the onasemnogene abeparvovec selectively induce SMN expression in the spinal cord or neural cells. Therefore, when performing peripheral blood analysis, the relationship between the target organs and the pharmacokinetics of these drugs must be considered.

### Validity of SMN spot analysis

In a previous report, we demonstrated that SMN spot analysis can reveal the aggregation of SMN proteins in the cell nucleus, as well as their association with functional molecules. In other words, we conclude that the significance of SMN spot analysis is not simply to compare the amount of SMN protein but also to monitor functional SMN expression. Prior to the umbilical cord blood study, we analysed 37 patients with SMA before DMT administration to demonstrate its reliability as a biomarker for the pathological condition of SMA (Fig. 1A–C). Spearman's rank correlation analysis showed that SMN spot analysis had a slightly stronger correlation with the *SMN2* copy number (*rs* > 0.4) than with the clinical classification of SMA (types I–III and Ia–IIIb, *rs* > 0.3). The Jonckheere-Terpstra analysis showed a similar increase, with significant differences (**p < 0.01, *p < 0.05). Although the clinical classification of SMA may be affected by the patient's living and medical environments until the confirm diagnosis, the *SMN2* copy number remains constant. In addition, the *SMN2* copy number often plays a role in determining SMN protein expression in patients. That may be why the *SMN2* copy number was slightly more correlated with SMN protein levels than the clinical classifications.

### SMN spot analysis value between carriers and patients

Analysis values for the carrier and patient groups were compared. In vitro experiments using a fibroblast cell line showed significant differences in SMN production of cultured cells or exosomes derived from the patient, carrier, and control [35]. However, the quantification of SMN in PBMC by ELISA showed no significant differences between the carrier and patient groups [30]. The latter authors attributed their results to two factors. Firstly, eight of the twelve cases in the patient group were children under four years old, while all 15 cases in the carrier group were adults between 25 and 57 years old. Second, four of the twelve patients were taking the histone deacetylase (HDAC) inhibitor valproic acid for the clinical trial, which may have increased SMN production. In 2022, a method was developed to evaluate *SMN* mRNA in PBMCs, using quantitative fluorescence real-time PCR (QF-RT-PCR) as a biomarker [36]. Their method calculated the percentage of FL-*SMN* transcripts among all *SMN*-related transcripts, rather than quantifying *SMN* expression. Significant differences were observed between the patient, carrier, and control groups.

In our SMN spot analysis, the carrier group showed significantly lower levels than the control group, although they were not patients. And we did not observe any significant differences between the carrier and patient group. The analysis values for the carrier group varied widely, ranging from 1.4 to 27.3% (Additional file 4AB), similar to those of the patient group ranging from 0.2 to 31.7% (Additional file 3C). The reasons for this are discussed below:

In this study, we did not investigate the copy numbers of *SMN1* and *SMN2* in the carriers. According to previous studies, 90% of the carriers are heterozygous for one copy of *SMN1* [37]. Owing to *SMN1* heterozygosity, carriers may tend to have lower systemic SMN expression, which may be reflected in the analytical values. For instance, in Glanzmann thrombasthenia, an autosomal recessive genetic disease, asymptomatic carriers who are heterozygous for the causative gene (*ITGA2B* or *ITGB3*) tend to have a lower expression of integrin glycoprotein IIb-IIIa on the platelet surface than those in the control group [38].

Another factor to consider is the presence of *SMN1* and *SMN2* variants. There are many variants of *SMN2*, 16 of which have been reported to positively or negatively modulate exon 7 splicing, even if they range from intron 6 to exon 8 [39]. Additionally, more than 80 variants have been reported for *SMN1*, and changes in SMN protein expression and function due to changes in functional sites such as the Tudor and C-terminal domains have been demonstrated [40]. Conformational changes can promote non-aggregation or disassembly of SMN, which can affect the results of our SMN spot analysis.

In our SMN spot analysis, all 18 participants in the carrier group were adults (32–48 years), whereas the 37 participants in the patient group included 17 adults (16–62 years) and 20 children (< 16 years). Although the composition of the patient group differed from that reported by Kobayashi et al., no significant differences were observed, similar to their report. On the other hand, an important aspect of QF-RT-PCR is to focus on the differences in the copy numbers of *SMN1* and *SMN2* in three groups: control, carrier, and patient, and to compare the production ratios of FL-*SMN* among the total *SMN* transcripts, including variants [36]. Their approach is similar to ours in that it involves a comparison of the functional ratio of total expression rather than a simple quantitative comparison. In their analysis, each group consisted of 31–44 participants; thus, we need to analyse more carriers.

To better understand the variability among carriers, we examined the relationship between the mother's analysis value and the SMA type of each child (see Fig. 1E, Additional file 4). Although it has not yet been firmly established, our unpublished data indicates a trend toward a decrease in SMN spot expression over approximately age 40 (data not shown). Excluding the two cases involving mothers over 40, the analysis of the eight mothers under 40 years old revealed a partial correlation between their analysis value and the severity of SMA in their children. This suggests that the analysis values from carrier mothers may indicate the severity of SMA in their children, or may vary based on the mother's age.

### Analysis of SMN expression correlation between foetuses and mothers

We discussed the correlation between SMN spot analysis in maternal peripheral blood and neonatal umbilical cord blood (Fig. 3). According to Spearman's rank correlation coefficient, the theoretical critical value for rejecting the null hypothesis with a sample size of five cases was *rs* = 1.00 (*p* < 0.05) [41]. The result shown in Fig. 3 was *rs* = 1.00 (*p* = 0.017), indicating a correlation between neonatal and maternal data. However, owing to the limited number of cases, only five pairs were analysed in this study. This article presents the results of the Z-score by participant group affiliation. The results are presented as SD to aid understanding, but the Robust Z-Score using the normalised interquartile range (NIQR, see Additional file 3) yields similar results. As in the statistical analysis presented above, the theoretical critical value for rejecting the null hypothesis with a sample size of eight cases is *rs* = 0.738 (*p* < 0.05) [41]. For the group of eight mothers under age 40, as indicated in Fig. 1E, was *rs* = 0.792 (*p* = 0.019), which meets the significance level. In this study, we could only detect a partial correlation for severe cases, excluding SMA type III, taking into account maternal age. Meanwhile, the current target of prenatal diagnosis is severe cases of SMA types 0 through II. Our results imply two possibilities for rapid therapeutic intervention: 1) the severity of SMA after birth can be predicted through the analysis of maternal peripheral blood, and 2) inducing maternal SMN expression could serve as an effective treatment strategy for the foetus. A larger sample size than that analysed in this study is necessary to confirm these findings.

### SMN dynamics during the foetal period

SMN expression is necessary during development, as *smn* deficient leads to embryonic lethality in mice [42]. In humans, the significance of paralogue of *SMN1* is also understood, as no patients with SMA have been found with zero copies of *SMN2* [43]. Studies have shown that SMN expression is increased during the embryonic and foetal stages in human tissues than during the postnatal period. However, obtaining sufficient spinal cord specimens from embryonic to postnatal stages is difficult, particularly in humans. Ramos et al.’s study in 2019 provided valuable insights into the quantification of SMN protein expression in the spinal and cerebral cortices of humans from 15 weeks of gestation to 14 years after birth [14]. The study showed that the period with the highest protein expression level was during the foetal period, specifically from the 15th to the 39th weeks of gestation, in 25 cases. The protein expression level decreased by half in 19 cases from birth to 3-month-old, and in 21 cases after 3-month-old, it decreased to approximately one-seventh of the foetal period. These findings suggest that the expression of the SMN protein is crucial during a limited period from 15 weeks of gestation to approximately 3 months after birth.

Here, we considered SMN dynamics during the embryonic period. Interestingly, even in defective *SMN1*, the foetus continued to grow and was born without any SMA symptoms. We considered the possibility that SMN is supplied to the foetus through both foetal own *SMN2* and maternal blood via the placenta. This was based on the correlation between the mother and neonate, as shown in Fig. 3.

Small molecules with a molecular weight of 1000 Da or less (usually between 300 and 600 Da) can pass from the mother to the foetus through the placenta. SMN has a molecular weight of 39 kDa, and there have been no reports of it crossing the placenta. However, recent studies have revealed that cell movement between the mother and foetus is necessary during the ontogenetic stages. Maternal microchimeric cells (MMc), in which maternal cells migrate to the foetus, occur before fertilised eggs are implanted [44]. A report indicated that MMc-derived the specific nucleotide was detected in umbilical cord blood during the second trimester of pregnancy [45]. Migration of MMc was observed in the foetal brain before the blood–brain barrier (BBB) function was completed, suggesting that it is involved in the construction of neural circuits in the foetus [46]. Pregnancy is established between the mother and embryo through cell–cell contact and communication between extracellular vesicles (EVs) and other soluble factors [47]. The EV cargo protein has been identified that activates foetal trophoblast cells and enhances decidualisation and angiogenesis via endometrial EVs [48]. Because SMN has also been reported to be an EV cargo protein [35], we hypothesised that foetal growth could be supported by SMN supplementation via maternal cells or cell-derived factors during pregnancy.

### The prospect of onset due to decreased SMN expression after birth

In general, even neonates with severe type I SMA do not show any clinical symptoms such as muscle weakness or hypotonia at birth. Usually, after one month of birth, infants become less active and their motor function are no longer out of synchronisation with their growth, which is considered to be the onset of SMA.

A study by Ramos et al. on the cerebrum and spinal cord of 75 non-SMA patients found that the mRNA expression frequency of FL-*SMN1* and FL-*SMN2* was the highest during the foetal period and decreased significantly after birth, similar to SMN production at the protein level. Specifically, FL-*SMN1* expression begins to decrease three months after birth, whereas FL-*SMN2* expression decreases significantly immediately after birth [14]. Infants with only *SMN2* will experience faster SMN loss. None of the five neonates in Case 1–5 developed SMA at birth, suggesting that even with zero copies of *SMN1* exons 7 and 8, the necessary quality and quantity of SMN protein was supplied during the foetal period.

The SMN spot analysis values decreased as the days passed in Case 3 and 5, which showed high values in the cord blood at birth. However, Cases 1, 2, and 4, whose values were already low at birth, exhibited no obvious decrease over time. The SMN spot analysis value in the peripheral blood of infants with SMA is expected to decrease gradually after birth. In other words, SMN protein levels decrease over time after birth and are eventually depleted in the spinal cord, leading to the onset of SMA.

Case 3 and 5 with high values in cord blood were females, and Cases 1, 2, and 4 with low values in cord blood were males (Table 1). Although we did not examine sex-specific protective modifiers like plastin 3 (PLS3) in this study, the results are intriguing [49, 50].

### Maternal-mediated foetal treatment strategies

In animal models, therapeutic strategies via the maternal blood for foetuses with the SMA have been developed. Non-surgical interventions in the foetus include oral administration of HDAC inhibitors to the mother [51, 52] and incorporation of low-molecular-weight *SMN2* splicing modifiers into the mother's feed [53]. These treatments have been found to prevent foetal lethality and extend survival after birth in severe SMA models.

When administering SMN (39KDa) to the foetus via the mother, the problem is that the molecular weight is large. This may be overcome by synthesizing a fusion protein, which is a human IgG Fc domain binding SMN protein, similar to blood coagulation factor VIII Fc (200–350KDa) [54]. The blood coagulation factor VIII Fc fusion protein could cross the placenta via neonatal Fc receptors (FcRn) expressed in the placenta [55]. Alternatively, low-molecular-weight SMN modifiers such as the already approved risdiplam can be administered (401 Da), which may cross the placenta and induce FL-*SMN2* expression.

Based on our results of starting DMT at approximately 40 weeks (37–41 weeks) of planned full-term birth, inducing SMN expression in the foetus via the mother may be beneficial even late in pregnancy. This timeline ensures the safety and efficacy of the mother and foetus.

We conducted a study on five cases of presymptomatic SMA for which a treatment plan was established prenatally with the goal of a full-term planned birth at approximately 40 weeks. Without prompt initiation of DMT, each of the five presymptomatic neonates would have followed a similar history of severe SMA in the firstborns [56]. Several discoveries have been made by analysing SMN spots in the blood of mothers and neonates, particularly in umbilical cord blood. In cases in which severe SMA is anticipated in the foetus, planned delivery offers would control the onset of SMA through rapid DMT intervention. Elucidation of SMN dynamics in mothers and foetuses will further clarify the basis of strategic treatment planning. Furthermore, in addition to the analysis of umbilical cord blood, continuous analysis of peripheral blood after birth enabled us to understand the gradual progression of SMA development. Our demonstration has also highlighted the practical importance of newborn screening for SMA. Then, it is crucial to obtain a definitive diagnosis resulting from genetic testing as soon as possible after screening and to link the diagnosis to DMT intervention quickly.

## Conclusions

We established a correlation between the SMN spot analysis and the severity of SMA and *SMN2* copy numbers. This finding suggests that SMN spot analysis may be a reliable biomarker for determining the therapeutic efficiency of SMA. Functional SMN expression analysis using cord blood revealed the possibility of estimating the state of the motor neuron pool. After birth, the SMN gradually decreases over time, and if the therapeutic intervention for SMA is delayed, the depletion of the SMN protein in the spinal cord will progress gradually and lead to the onset of SMA within a few weeks. Moreover, we found a correlation between the SMN spot analysis values of the neonate's umbilical cord blood and the mother's peripheral blood, indicating that the SMN spot may predict the severity of SMA in the foetus from the mother's peripheral blood. The SMN derived from the mother may also be supplied to the foetus. Our findings provide a rationale for prompt treatment initiation for SMA and the efficacy of maternal-mediated treatment strategies. As a practical issue, this study emphasizes the importance of newborn screening for SMA. By establishing an intervention system in which DMT can be started immediately after newborn screening and a definitive diagnosis through genetic testing, it is possible to suppress the onset of the SMA and develop motor function.

## Supplementary Information

Additional file 1.

Additional file 2.

Additional file 3.

Additional file 4.

## Data Availability

The datasets used and/or analysed in the current study are available from the corresponding author upon reasonable request.

## Abbreviations

SMA: Spinal muscular atrophy
SMN: Survival motor neuron
DMT: Disease-modifying therapy
IFC: Imaging flow cytometry
PBMC: Peripheral blood mononuclear cell
mAb: Monoclonal antibody
FL-: Full length-
SD: Standard deviation
NIQR: Normalised interquartile range
DTR: Deep tendon reflexes
HDAC: Histone deacetylase
ITGA2B: Integrin subunit alpha 2b
ITGB3: Integrin beta 3
NF: Neurofilament
Nf-L: Neurofilament light chain
pNf-H: Phosphorylated neurofilament heavy chain
ELISA: Enzyme-linked immunosorbent assay
HTRF: Homogeneous time resolved fluorescence
ECL: Electrochemiluminescence
QF-RT-PCR: Quantitative fluorescence real-time polymerase chain reaction
PLS 3: Plastine 3
FcRn: Neonatal Fc receptor
MMc: Maternal microchimeric cells
EV: Extracellular vesicle
BBB: Blood–brain barrier

## References

[1] Lefebvre S, Bürglen L, Reboullet S, Clermont O, Burlet P, Viollet L, et al. Identification and characterization of a spinal muscular atrophy-determining gene. Cell. 1995;80(1):155–65. 10.1016/0092-8674(95)90460-3. 7813012 10.1016/0092-8674(95)90460-3

[2] Zerres K, Rudnik-Schöneborn S. Natural history in proximal spinal muscular atrophy. Clinical analysis of 445 patients and suggestions for a modification of existing classifications. Arch Neurol. 1995;52(5):518–23. 10.1001/archneur.1995.00540290108025. 7733848 10.1001/archneur.1995.00540290108025

[3] Faravelli I, Riboldi GM, Rinchetti P, Lotti F. The SMN complex at the crossroad between RNA metabolism and neurodegeneration. Int J Mol Sci. 2023;24:3. 10.3390/ijms24032247. 10.3390/ijms24032247 PMC9917330 36768569

[4] Broccolini A, Engel WK, Askanas V. Localization of survival motor neuron protein in human apoptotic-like and regenerating muscle fibers, and neuromuscular junctions. NeuroReport. 1999;10(8):1637–41. 10.1097/00001756-199906030-00003. 10501549 10.1097/00001756-199906030-00003

[5] Kim JK, Jha NN, Feng Z, Faleiro MR, Chiriboga CA, Wei-Lapierre L, et al. Muscle-specific SMN reduction reveals motor neuron-independent disease in spinal muscular atrophy models. J Clin Invest. 2020;130(3):1271–87. 10.1172/jci131989. 32039917 10.1172/JCI131989 PMC7269591

[6] Tisdale S, Van Alstyne M, Simon CM, Mentis GZ, Pellizzoni L. SMN controls neuromuscular junction integrity through U7 snRNP. Cell Rep. 2022;40(12):111393. 10.1016/j.celrep.2022.111393. 36130491 10.1016/j.celrep.2022.111393 PMC9533342

[7] Lorson CL, Hahnen E, Androphy EJ, Wirth B. A single nucleotide in the SMN gene regulates splicing and is responsible for spinal muscular atrophy. Proc Natl Acad Sci U S A. 1999;96(11):6307–11. 10.1073/pnas.96.11.6307. 10339583 10.1073/pnas.96.11.6307 PMC26877

[8] Monani UR, Lorson CL, Parsons DW, Prior TW, Androphy EJ, Burghes AH, et al. A single nucleotide difference that alters splicing patterns distinguishes the SMA gene SMN1 from the copy gene SMN2. Hum Mol Genet. 1999;8(7):1177–83. 10.1093/hmg/8.7.1177. 10369862 10.1093/hmg/8.7.1177

[9] Lunn MR, Wang CH. Spinal muscular atrophy. Lancet. 2008;371(9630):2120–33. 10.1016/S0140-6736(08)60921-6. 18572081 10.1016/S0140-6736(08)60921-6

[10] Lefebvre S, Burlet P, Liu Q, Bertrandy S, Clermont O, Munnich A, et al. Correlation between severity and SMN protein level in spinal muscular atrophy. Nat Genet. 1997;16(3):265–9. 10.1038/ng0797-265. 9207792 10.1038/ng0797-265

[11] Babić M, Banović M, Berečić I, Banić T, Babić Leko M, Ulamec M, et al. Molecular biomarkers for the diagnosis, prognosis, and pharmacodynamics of spinal muscular atrophy. J Clin Med. 2023. 10.3390/jcm12155060. 10.3390/jcm12155060 PMC10419842 37568462

[12] Glascock J, Darras BT, Crawford TO, Sumner CJ, Kolb SJ, DiDonato C, et al. Identifying biomarkers of spinal muscular atrophy for further development. J Neuromuscul Dis. 2023;10(5):937–54. 10.3233/JND-230054. 37458045 10.3233/JND-230054 PMC10578234

[13] Otsuki N, Arakawa R, Kaneko K, Aoki R, Arakawa M, Saito K. A new biomarker candidate for spinal muscular atrophy: Identification of a peripheral blood cell population capable of monitoring the level of survival motor neuron protein. PLoS ONE. 2018;13(8):e0201764. 10.1371/journal.pone.0201764. 30102724 10.1371/journal.pone.0201764 PMC6089418

[14] Ramos DM, d’Ydewalle C, Gabbeta V, Dakka A, Klein SK, Norris DA, et al. Age-dependent SMN expression in disease-relevant tissue and implications for SMA treatment. J Clin Invest. 2019;129(11):4817–31. 10.1172/jci124120. 31589162 10.1172/JCI124120 PMC6819103

[15] Lee BH, Waldrop MA, Connolly AM, Ciafaloni E. Time is muscle: a recommendation for early treatment for preterm infants with spinal muscular atrophy. Muscle Nerve. 2021;64(2):153–5. 10.1002/mus.27261. 33959970 10.1002/mus.27261

[16] Nigro E, Grunebaum E, Kamath B, Licht C, Malcolmson C, Jeewa A, et al. Case report: a case of spinal muscular atrophy in a preterm infant: risks and benefits of treatment. Front Neurol. 2023;14:1230889. 10.3389/fneur.2023.1230889. 37780708 10.3389/fneur.2023.1230889 PMC10539898

[17] Kaneko K, Arakawa R, Urano M, Aoki R, Saito K. Relationships between long-term observations of motor milestones and genotype analysis results in childhood-onset Japanese spinal muscular atrophy patients. Brain Dev. 2017;39(9):763–73. 10.1016/j.braindev.2017.04.018. 28601407 10.1016/j.braindev.2017.04.018

[18] Kanda Y. Investigation of the freely available easy-to-use software ‘EZR’ for medical statistics. Bone Marrow Transplant. 2013;48(3):452–8. 10.1038/bmt.2012.244. 23208313 10.1038/bmt.2012.244 PMC3590441

[19] Faul F, Erdfelder E, Buchner A, Lang AG. Statistical power analyses using G*Power 3.1: tests for correlation and regression analyses. Behav Res Methods. 2009;41(4):1149–60. 10.3758/BRM.41.4.1149. 19897823 10.3758/BRM.41.4.1149

[20] Fundaun J, Kolski M, Molina-Álvarez M, Baskozos G, Schmid AB. Types and concentrations of blood-based biomarkers in adults with peripheral neuropathies: a systematic review and meta-analysis. JAMA Netw Open. 2022;5(12):e2248593. 10.1001/jamanetworkopen.2022.48593. 36574244 10.1001/jamanetworkopen.2022.48593 PMC9857490

[21] Ning L, Wang B. Neurofilament light chain in blood as a diagnostic and predictive biomarker for multiple sclerosis: a systematic review and meta-analysis. PLoS ONE. 2022;17(9):e0274565. 10.1371/journal.pone.0274565. 36103562 10.1371/journal.pone.0274565 PMC9473405

[22] Darras BT, Crawford TO, Finkel RS, Mercuri E, De Vivo DC, Oskoui M, et al. Neurofilament as a potential biomarker for spinal muscular atrophy. Ann Clin Transl Neurol. 2019;6(5):932–44. 10.1002/acn3.779. 31139691 10.1002/acn3.779 PMC6530526

[23] Olsson B, Alberg L, Cullen NC, Michael E, Wahlgren L, Kroksmark AK, et al. NFL is a marker of treatment response in children with SMA treated with nusinersen. J Neurol. 2019;266(9):2129–36. 10.1007/s00415-019-09389-8. 31123861 10.1007/s00415-019-09389-8 PMC6687695

[24] Totzeck A, Stolte B, Kizina K, Bolz S, Schlag M, Thimm A, et al. Neurofilament heavy chain and tau protein are not elevated in cerebrospinal fluid of adult patients with spinal muscular atrophy during loading with nusinersen. Int J Mol Sci. 2019. 10.3390/ijms20215397. 10.3390/ijms20215397 PMC6862027 31671515

[25] Walter MC, Wenninger S, Thiele S, Stauber J, Hiebeler M, Greckl E, et al. Safety and treatment effects of nusinersen in longstanding adult 5q-SMA Type 3—a prospective observational study. J Neuromuscul Dis. 2019;6(4):453–65. 10.3233/JND-190416. 31594243 10.3233/JND-190416 PMC6918909

[26] Wurster CD, Günther R, Steinacker P, Dreyhaupt J, Wollinsky K, Uzelac Z, et al. Neurochemical markers in CSF of adolescent and adult SMA patients undergoing nusinersen treatment. Ther Adv Neurol Disord. 2019;12:1756286419846058. 10.1177/1756286419846058. 31205491 10.1177/1756286419846058 PMC6535708

[27] Wurster CD, Steinacker P, Günther R, Koch JC, Lingor P, Uzelac Z, et al. Neurofilament light chain in serum of adolescent and adult SMA patients under treatment with nusinersen. J Neurol. 2020;267(1):36–44. 10.1007/s00415-019-09547-y. 10.1007/s00415-019-09547-y 31552549

[28] Faravelli I, Meneri M, Saccomanno D, Velardo D, Abati E, Gagliardi D, et al. Nusinersen treatment and cerebrospinal fluid neurofilaments: an explorative study on Spinal Muscular Atrophy type 3 patients. J Cell Mol Med. 2020;24(5):3034–9. 10.1111/jcmm.14939. 32032473 10.1111/jcmm.14939 PMC7077557

[29] Kobayashi DT, Olson RJ, Sly L, Swanson CJ, Chung B, Naryshkin N, et al. Utility of survival motor neuron ELISA for spinal muscular atrophy clinical and preclinical analyses. PLoS ONE. 2011;6(8):e24269. 10.1371/journal.pone.0024269. 21904622 10.1371/journal.pone.0024269 PMC3164180

[30] Kobayashi DT, Decker D, Zaworski P, Klott K, McGonigal J, Ghazal N, et al. Evaluation of peripheral blood mononuclear cell processing and analysis for Survival Motor Neuron protein. PLoS ONE. 2012;7(11):e50763. 10.1371/journal.pone.0050763. 23226377 10.1371/journal.pone.0050763 PMC3511312

[31] Naryshkin NA, Weetall M, Dakka A, Narasimhan J, Zhao X, Feng Z, et al. Motor neuron disease SMN2 splicing modifiers improve motor function and longevity in mice with spinal muscular atrophy. Science. 2014;345(6197):688–93. 10.1126/science.1250127. 25104390 10.1126/science.1250127

[32] Kolb SJ, Coffey CS, Yankey JW, Krosschell K, Arnold WD, Rutkove SB, et al. Baseline results of the NeuroNEXT spinal muscular atrophy infant biomarker study. Ann Clin Transl Neurol. 2016;3(2):132–45. 10.1002/acn3.283. 26900585 10.1002/acn3.283 PMC4748311

[33] Zaworski P, von Herrmann KM, Taylor S, Sunshine SS, McCarthy K, Risher N, et al. SMN protein can be reliably measured in whole blood with an electrochemiluminescence (ECL) immunoassay: implications for clinical trials. PLoS ONE. 2016;11(3):e0150640. 10.1371/journal.pone.0150640. 26953792 10.1371/journal.pone.0150640 PMC4783032

[34] Poirier A, Weetall M, Heinig K, Bucheli F, Schoenlein K, Alsenz J, et al. Risdiplam distributes and increases SMN protein in both the central nervous system and peripheral organs. Pharmacol Res Perspect. 2018;6(6):e00447. 10.1002/prp2.447. 30519476 10.1002/prp2.447 PMC6262736

[35] Nash LA, McFall ER, Perozzo AM, Turner M, Poulin KL, De Repentigny Y, et al. Survival motor neuron protein is released from cells in exosomes: a potential biomarker for spinal muscular atrophy. Sci Rep. 2017;7(1):13859. 10.1038/s41598-017-14313-z. 29066780 10.1038/s41598-017-14313-z PMC5655039

[36] Maretina M, Egorova A, Lanko K, Baranov V, Kiselev A. Evaluation of mean percentage of full-length SMN transcripts as a molecular biomarker of spinal muscular atrophy. Genes (Basel). 2022. 10.3390/genes13101911. 10.3390/genes13101911 PMC9602009 36292797

[37] Wirth B, Schmidt T, Hahnen E, Rudnik-Schöneborn S, Krawczak M, Müller-Myhsok B, et al. De novo rearrangements found in 2% of index patients with spinal muscular atrophy: mutational mechanisms, parental origin, mutation rate, and implications for genetic counseling. Am J Hum Genet. 1997;61(5):1102–11. 10.1086/301608. 9345102 10.1086/301608 PMC1716038

[38] Kashiwagi H, Kunishima S, Kiyomizu K, Amano Y, Shimada H, Morishita M, et al. Demonstration of novel gain-of-function mutations of αIIbβ3: association with macrothrombocytopenia and glanzmann thrombasthenia-like phenotype. Mol Genet Genomic Med. 2013;1(2):77–86. 10.1002/mgg3.9. 24498605 10.1002/mgg3.9 PMC3865572

[39] Costa-Roger M, Blasco-Pérez L, Cuscó I, Tizzano EF. The importance of digging into the genetics of SMN genes in the therapeutic scenario of spinal muscular atrophy. Int J Mol Sci. 2021. 10.3390/ijms22169029. 10.3390/ijms22169029 PMC8396600 34445733

[40] Qu YJ, Bai JL, Cao YY, Wang H, Jin YW, Du J, et al. Mutation spectrum of the survival of motor neuron 1 and functional analysis of variants in chinese spinal muscular atrophy. J Mol Diagn. 2016;18(5):741–52. 10.1016/j.jmoldx.2016.05.004. 27425821 10.1016/j.jmoldx.2016.05.004

[41] Pestman WR, Backmatter. In Mathematical statistics. De Gruyter, Berlin, 2009;585. 10.1515/9783110208535.

[42] Schrank B, Götz R, Gunnersen JM, Ure JM, Toyka KV, Smith AG, et al. Inactivation of the survival motor neuron gene, a candidate gene for human spinal muscular atrophy, leads to massive cell death in early mouse embryos. Proc Natl Acad Sci U S A. 1997;94(18):9920–5. 10.1073/pnas.94.18.9920. 9275227 10.1073/pnas.94.18.9920 PMC23295

[43] Tizzano E. Spinal muscular atrophy during human development: where are the early pathogenic findings? Adv Exp Med Biol. 2009;652:225–35. 10.1007/978-90-481-2813-6_15. 20225029 10.1007/978-90-481-2813-6_15

[44] Beal JR, Ma Q, Bagchi IC, Bagchi MK. Role of endometrial extracellular vesicles in mediating cell-to-cell communication in the uterus: a review. Cells. 2023. 10.3390/cells12222584. 10.3390/cells12222584 PMC10670844 37998319

[45] Kanaan SB, Gammill HS, Harrington WE, De Rosa SC, Stevenson PA, Forsyth AM, et al. Maternal microchimerism is prevalent in cord blood in memory T cells and other cell subsets, and persists post-transplant. Oncoimmunology. 2017;6(5):e1311436. 10.1080/2162402X.2017.1311436. 28638735 10.1080/2162402X.2017.1311436 PMC5467984

[46] Schepanski S, Chini M, Sternemann V, Urbschat C, Thiele K, Sun T, et al. Pregnancy-induced maternal microchimerism shapes neurodevelopment and behavior in mice. Nat Commun. 2022;13(1):4571. 10.1038/s41467-022-32230-2. 35931682 10.1038/s41467-022-32230-2 PMC9356013

[47] Bridi A, Perecin F, Silveira JCD. Extracellular vesicles mediated early embryo-maternal interactions. Int J Mol Sci. 2020. 10.3390/ijms21031163. 10.3390/ijms21031163 PMC7037557 32050564

[48] Ma Q, Beal JR, Bhurke A, Kannan A, Yu J, Taylor RN, et al. Extracellular vesicles secreted by human uterine stromal cells regulate decidualization, angiogenesis, and trophoblast differentiation. Proc Natl Acad Sci U S A. 2022;119(38):e2200252119. 10.1073/pnas.2200252119. 36095212 10.1073/pnas.2200252119 PMC9499590

[49] Oprea GE, Kröber S, McWhorter ML, Rossoll W, Müller S, Krawczak M, et al. Plastin 3 is a protective modifier of autosomal recessive spinal muscular atrophy. Science. 2008;320(5875):524–7. 10.1126/science.1155085. 18440926 10.1126/science.1155085 PMC4908855

[50] Yanyan C, Yujin Q, Jinli B, Yuwei J, Hong W, Fang S. Correlation of PLS3 expression with disease severity in children with spinal muscular atrophy. J Hum Genet. 2014;59(1):24–7. 10.1038/jhg.2013.111. 24172247 10.1038/jhg.2013.111

[51] Chang JG, Hsieh-Li HM, Jong YJ, Wang NM, Tsai CH, Li H. Treatment of spinal muscular atrophy by sodium butyrate. Proc Natl Acad Sci U S A. 2001;98(17):9808–13. 10.1073/pnas.171105098. 11504946 10.1073/pnas.171105098 PMC55534

[52] Riessland M, Ackermann B, Förster A, Jakubik M, Hauke J, Garbes L, et al. SAHA ameliorates the SMA phenotype in two mouse models for spinal muscular atrophy. Hum Mol Genet. 2010;19(8):1492–506. 10.1093/hmg/ddq023. 20097677 10.1093/hmg/ddq023

[53] Butchbach ME, Singh J, Gurney ME, Burghes AH. The effect of diet on the protective action of D156844 observed in spinal muscular atrophy mice. Exp Neurol. 2014;256:1–6. 10.1016/j.expneurol.2014.03.005. 24681157 10.1016/j.expneurol.2014.03.005 PMC4029929

[54] Powell JS, Josephson NC, Quon D, Ragni MV, Cheng G, Li E, et al. Safety and prolonged activity of recombinant factor VIII Fc fusion protein in hemophilia A patients. Blood. 2012;119(13):3031–7. 10.1182/blood-2011-09-382846. 22223821 10.1182/blood-2011-09-382846 PMC3646317

[55] Mimoun A, Bou-Jaoudeh M, Delignat S, Daventure V, Reyes Ruiz A, Lecerf M, et al. Transplacental delivery of therapeutic proteins by engineered immunoglobulin G: a step toward perinatal replacement therapy. J Thromb Haemost. 2023. 10.1016/j.jtha.2023.05.021. 10.1016/j.jtha.2023.05.021 37271431

[56] Jones CC, Cook SF, Jarecki J, Belter L, Reyna SP, Staropoli J, et al. Spinal muscular atrophy (SMA) subtype concordance in siblings: findings from the cure SMA cohort. J Neuromuscul Dis. 2020;7(1):33–40. 10.3233/JND-190399. 31707372 10.3233/JND-190399 PMC7029365

