# Supplementary material for: Analysis of SMN protein in umbilical cord blood and postnatal peripheral blood of neonates with SMA: a rationale for prompt treatment initiation to prevent SMA development
Source: Orphanet J Rare Dis. 2025 Feb 28;20:91. doi: 10.1186/s13023-025-03597-4 (PMC11869478; doi:10.1186/s13023-025-03597-4)
Supplement: Supplementary file 1 — Additional file 1. [file 13023_2025_3597_MOESM1_ESM.pdf]

Additional file 1.
Analytical data and information of 37 spinal muscular atrophy (SMA) patients.
Details of data in Figure 1 (A-C)

ID	Sex	Age	SMA Typing	*SMN2* (Copies)	SMN-Spot[+] cells (%)
S1	M	1m12d	Ia	2	3.3
S2	M	1m16d	Ia	2	1.6
S3	M	2m1d	Ia	2	0.4
S4	M	2m26d	Ia	2	1.2
S5	M	1y10m	Ia	2	1.1
S6	M	5y3m	Ib	2	12.4
S7	M	24y7m	IIa	2	14.5
S8	M	45y9m	IIa	2	4.3
S9	M	8y9m	IIb	2	13.8
S10	M	13y11m	IIb	2	1.1
S11	F	16y3m	IIb	2	7.8
S12	F	19y2m	IIb	2	4.9
S13	F	29y1m	IIIa	2	14.7
S14	F	10m23d	Ib	3	10.5
S15	F	11m5d	Ib	3	2.6
S16	M	1y1m	Ib	3	12.3
S17	M	1y10m	Ib	3	19.6
S18	M	2y4m	IIa	3	3.5
S19	F	25y0m	IIa	3	4.4
S20	F	33y8m	IIa	3	11.0
S21	M	51y1m	IIa	3	8.9
S22	F	11y10m	IIb	3	16.2
S23	M	14y5m	IIb	3	0.2
S24	F	19y10m	IIb	3	14.4
S25	M	23y11m	IIb	3	6.1
S26	M	3y3m	IIIa	3	4.5
S27	M	8y2m	IIIa	3	8.3
S28	F	14y8m	IIIa	3	4.5
S29	F	17y11m	IIIa	3	31.7
S30	M	35y3m	IIIb	3	15.2
S31	M	48y7m	IIIb	3	0.5
S32	M	8y0m	IIIb	4	18.8
S33	M	11y5m	IIIb	4	7.9
S34	F	16y4m	IIIb	4	14.8
S35	F	21y3m	IIIb	4	16.3
S36	F	44y0m	IIIb	4	8.3
S37	F	62y3m	IIIb	4	19.2
