# Supplementary material for: Analysis of SMN protein in umbilical cord blood and postnatal peripheral blood of neonates with SMA: a rationale for prompt treatment initiation to prevent SMA development
Source: Orphanet J Rare Dis. 2025 Feb 28;20:91. doi: 10.1186/s13023-025-03597-4 (PMC11869478; doi:10.1186/s13023-025-03597-4)
Supplement: Supplementary file 2 — Additional file 2. [file 13023_2025_3597_MOESM2_ESM.pdf]

Additional file 2.

Analysis of survival motor neuron (SMN) spot-positive cells in the population strongly positive for CD33 ($CD33^{++}$) cells

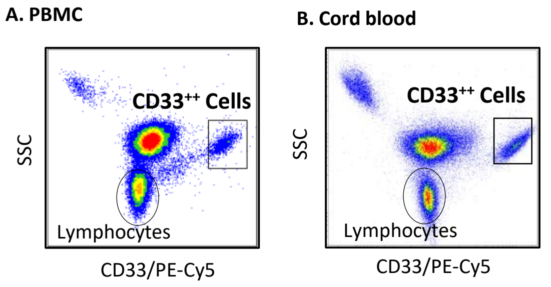

The target fraction of survival motor neuron (SMN) spot analysis

Peripheral blood nuclear cells (PBMC) and (B) umbilical cord blood (cord blood) were analyzed using anti-CD33 mAb (x-axis) and side scatter (SSC; y-axis). $CD33^{++}$ cells are marked with squares.

The detail of this fraction and SMN-staining images are described in [13].
