# Supplementary material for: Analysis of SMN protein in umbilical cord blood and postnatal peripheral blood of neonates with SMA: a rationale for prompt treatment initiation to prevent SMA development
Source: Orphanet J Rare Dis. 2025 Feb 28;20:91. doi: 10.1186/s13023-025-03597-4 (PMC11869478; doi:10.1186/s13023-025-03597-4)
Supplement: Supplementary file 3 — Additional file 3. [file 13023_2025_3597_MOESM3_ESM.pdf]

Additional file 3.
Statistical analysis by the classifications of 37 spinal muscular atrophy (SMA) patients
(A) SMA typing, (B) SMA subtype, and (C) copy number of *SMN2*. Details of data in Figure 1 (A-C).

A.

**SMA typing**	Type I	Type II	Type III
n	10	14	13
% SMN-spot+ cells Mean	6.5	7.9	12.7
**SD**	6.7	5.3	8.2
RSD (%)	102.7	66.7	64.9
**% SMN-spot+ cells**			
Min	0.4	0.2	0.5
Quartile 1	1.3	4.3	7.9
**Median**	2.9	6.9	14.7
Quartile 3	11.9	13.1	16.3
Max	19.6	16.2	31.7
IQR *a)*	10.6	8.8	8.4
**NIQR** *b)*	7.8	6.5	6.3

a) IQR; Interquartile range (Q3-Q1) *b*) NIQR; Normalized IQR (IQR/1.3489)

B.

**SMA subtype**	Type I		Type II		Type III	
	a	b	a	b	a	b
n	5	5	5	9	5	8
% SMN-spot+ cells Mean (%)	1.5	11.5	8.6	7.6	12.7	12.6
**SD**	1.1	6.1	4.4	5.9	11.4	6.5
RSD (%)	71.0	52.8	50.9	78.7	89.3	51.3
**% SMN-spot+ cells**						
Min	0.4	2.6	4.3	0.2	4.5	0.5
Quartile 1	1.1	10.5	4.4	3.5	4.5	8.2
**Median**	1.2	12.3	8.9	6.1	8.3	15.0
Quartile 3	1.6	12.4	11.0	13.8	14.7	16.9
Max	3.3	19.6	14.5	16.2	31.7	19.2
IQR *a)*	0.6	1.9	6.6	10.2	10.2	8.7
**NIQR** *b)*	0.4	1.4	4.9	7.6	7.5	6.5

a) IQR; Interquartile range (Q3-Q1) *b*) NIQR; Normalized IQR (IQR/1.3489)

C.

***SMN2* copy number**	SMA				Non-SMA	
	2 copies	3 copies	4 copies	Total	Carriers	Controls
n	13	18	6	37	18	14
% SMN-spot^{+} cells Mean (%)	6.2	9.7	14.2	9.2	9.0	31.0
**SD**	5.7	7.8	5.0	7.1	8.5	8.7
RSD (%)	91.0	81.0	35.2	77.4	93.9	28.2
**% SMN-spot^{+} cells**						
Min	0.4	0.2	7.9	0.2	1.4	13.2
Quartile 1	1.2	4.4	10.0	3.5	3.0	27.0
**Median**	4.3	8.6	15.6	8.3	5.1	31.0
Quartile 3	12.4	13.9	18.2	14.5	14.4	36.6
Max	14.7	31.7	19.2	31.7	27.3	43.0
IQR [a)]	11.2	9.5	8.2	11.0	11.4	9.6
**NIQR** [b)]	8.3	7.0	6.1	8.1	8.4	7.1

a) IQR; Interquartile range (Q3-Q1)   *b*) NIQR; Normalized IQR (IQR/1.3489)
