# Supplementary material for: Analysis of SMN protein in umbilical cord blood and postnatal peripheral blood of neonates with SMA: a rationale for prompt treatment initiation to prevent SMA development
Source: Orphanet J Rare Dis. 2025 Feb 28;20:91. doi: 10.1186/s13023-025-03597-4 (PMC11869478; doi:10.1186/s13023-025-03597-4)
Supplement: Supplementary file 4 — Additional file 4. [file 13023_2025_3597_MOESM4_ESM.pdf]

Additional file 4.
Analytical data and information of 18 spinal muscular atrophy (SMA) carriers and clinical information of their children.
(A) Mothers (ten) and (B) fathers (eight). Details of data in Figure 1D and E.

A. Carrier (Female)

		Children with SMA	
Age	SMN-Spot$^+$ cells (%)	*SMN2* (Copies)	SMA Typing
32	1.4	2	0
25	2.5	2	Ia
38	3.4	2	Ia
39	2.7	3	Ib
39	6.9	3	Ib
33	16.4	3	Ib
35	25.2	3	Ib
39	20.1	2	IIb
42	5.0	3	IIIa
47	7.4	4	IIIb

B. Carrier (Male)

		Children with SMA	
Age	SMN-Spot$^+$ cells (%)	*SMN2* (Copies)	SMA Typing
32	1.5	2	0
39	27.3	2	Ia
44	5.1	3	Ib
38	3.3	3	Ib
46	8.4	3	Ib
38	18.9	3	Ib
41	2.9	3	IIIa
46	4.6	4	IIIb
